# State Board of Health Bill

**Published:** 1899-03

**Authors:** 


### EDITORIAL DEPARTMENT.

F. E. DANIEL, M. D., Editor.
S. E. HUDSON, M. D., Managing Editor.
A. J. SMITH. M. D., Galveston, Associate Editor.

Published Monthly at Austin, Texas, by Drs. Daniel and Hudson. Subscription price $1.00 a year in advance.

Eastern Representative: John Guy Monihan, St. Paul Building, 220 Broadway New York City.

Official organ of the West Texas Medical Association, the Houston District Medical Association, the Austin District Medical Society, the Brazos Valley Medical Association, the Galveston County Medical Society, and several others.

### State Board of Health Bill.

WE GIVE BELOW the draft of a bill to create a State Board of Health, as prepared by the State Medical Association's committee, (of which Dr. R. H. Harrison, Sr., of Columbus, Texas, is chairman, with Drs. Red of Houston, Saunders of Fort Worth, Osborn of Cleburne and M. M. Smith of Austin as members). The bill was introduced in the Senate on the 10th inst., by Senator Kerr, and on account of the proposed "penalties" was referred to the Judiciary Committee (Senator Yantis, chairman) instead of to the Committee on Public Health. We are not advised that Governor Sayers has recommended any sanitary legislation, as the medical profession had been led to hope he would do, from statements made by his friends. The title of the bill, in my opinion, is unfortunate. It should have been called "An Act to provide for additional security to the public health," or something of that sort, inasmuch as the mere mention of a "medical board" seems to have the same effect on some law makers hereabout that a red rag has on a bull; it arouses an antagonism *per se,* and they will hear no arguments.

The writer does not share the hope which seems to animate the committee. Esau seems to be wedded to his quarantine idol, and we had as well let him alone until the question of State sanitation can be made, by the 5000 physicians of Texas, a factor in the election or defeat of candidates for the Legislature.

### BILL TO CREATE A STATE BOARD OF HEALTH, DEFINE ITS DUTIES AND POWERS, AND PROVIDE FOR ITS MAINTENANCE IN THE STATE OF TEXAS.

### PREAMBLE.

WHEREAS, The great extent of territory, rapid increase in population, and vast commercial importance of the State of Texas requires the highest grade of intelligence, and keenest watchfulness for the preservation of the health as well as the property of the people; and

WHEREAS, The past few years have fully demonstrated the necessity for a more expansive system of sanitation than any that has ever prevailed in Texas before; and since most of the States in the Union having interest in the matter of public health have long since taken the initiative in this respect and moved abreast of the times in the matter of advanced modern sanitation, therefore, be it enacted by the Legislature of the State of Texas, that

ARTICLE 1. There shall be created a "State Board of Health" consisting of seven physicians, to be selected on account of their experience and fitness, five of whom from the five supreme judicial districts of the State, and two from the State at large, whose methods of appointment, terms of office, duties, powers and recompense shall be prescribed in the following articles.

### HOW FORMED.

ARTICLE 2, SECTION 1. Said State Board of Health shall be appointed by the Governor of Texas and confirmed by the Senate as soon as practicable after the passage of this act, and each member thereof shall serve for a period of six years or until his successor shall have qualified, except in the creation of the first board one shall be appointed for one year, one member for two years, one member for three years, etc., and as a vacancy occurs annually, or at any other time, it will be the duty of the Governor to fill the same by appointing a member to serve an unexpired term or for six years.

SECTION 2. As soon as practicable after their appointment, said State Board of Health shall convene and elect one of their members to be president, who will be the "chief health officer" of Texas, whose duty it shall be to act for the board in all emergencies requiring the prompt administration or other duty until it can convene and take action. It shall also elect a secretary and other such officers and assistants as may be necessary to carry out successfully all objects of the board.

SECTION 3. Said State Board of Health shall hold its regular meetings every six months, or special ones oftener if necessary, at the capitol and in a room to be furnished by the State. They shall own a seal, and be required to make a report of their doings, annually, to the Governor of Texas.

### DUTIES.

ARTICLE 3, SECTION 1. The duties of the State Board of Health shall be to take full cognizance of everything that pertains to the public health in the State of Texas. They shall promptly inquire into all epidemic and dangerous diseases which may threaten human life in the State, and do all in their power to abate them. They shall take charge of all quarantines between this State and all outside localities, and shall institute and abolish the same at their discretion. They shall appoint such officers, guards and inspectors as may be necessary to protect the public health in time of danger; establish their salaries, and do all things not incompatible with the laws of Texas in averting epidemics and diseases of a dangerous character.

SECTION 2. They shall take cognizance of all articles of food offered for sale in the State of Texas, as likewise all drugs and all other articles consumed by man, to the end that nothing but pure, healthful and unadulterated articles of food and drink be sold in said State to any citizen or resident thereof.

SECTION 3. The State Board of Health shall investigate the condition of the insane asylums and all other State, county and municipal charities. They shall inquire into the causes of diseases, and by careful study and advice to the proper authority, endeavor to diminish the number and ameliorate the condition of such cases.

SECTION 4. Said board shall prepare a system of vital statistics, collect a list of all deaths and births in the State of Texas, when practicable, and distribute same at home and abroad, as they may deem proper for the dissemination of sanitary knowledge and the advantage of the State.

SECTION 5. The State Board of Health shall inquire into all epidemic diseases occurring among horses and cattle, and enforce such laws and regulations as to them may seem necessary in order to abate such diseases.

### POWERS OF THE BOARD.

ARTICLE 4, SECTION 1. For the purpose of carrying out the foregoing duties the Board of Health shall be empowered to secure the services of a competent chemist and bacteriologist, veterinary surgeon, and also such guards, inspectors and other assistants as may be necessary to fulfill the objects of this act, whose salaries may be determined by their by-laws.

Section 2. They shall appoint all State quarantine officers, define their respective duties, and arrange for their recompense.

Section 3. To institute and abolish all quarantines between this and other States, and to regulate all interior quarantines.

Section 4. To control, limit and suppress all contagious and infectious diseases within the State, whether occurring amongst men or animals.

Section 6. Full authority to make and enforce such rules and regulations not inconsistent with the laws of Texas as may be necessary to carry out the objects of their creation.

Section 7. They shall have power to detain and inspect vessels, trains, persons, animals and other things when deemed infected or suspected with dangerous disease, and to forward same when free from danger. They shall furnish same with a "certificate of health," if found in perfect sanitary condition, and such certificate of health shall entitle the holder thereof to uninterrupted passage through the State, unless such certificate of health is revoked by the authority that caused its issuance.

Section 8. Any person or persons who shall interrupt or detain any such person or thing so released by said Board of Health and holding its proper certificate of health, unless such certificate of health is revoked by the authority who caused its issuance, shall be guilty of a misdemeanor, and upon conviction shall be punished by a fine of not less than $50 nor more than $100 each and imprisonment at the discretion of the court.

Section 9. In time of epidemics in the State, said board shall have authority to use signals on railroad trains to designate their freedom from dangerous disease, and such signals when displayed shall entitle such train to stop at or proceed by any station on its line, unless such certificate of health is revoked by the authority that caused its issuance.

Section 10. Any person interrupting trains bearing such signals will be guilty of a misdemeanor and liable to a fine of $100 for every such offense.

Section 11. Any person who shall display a signal or a certificate of health not authorized by the State Board of Health for the purpose of eluding detention or inspection, will be guilty of a felony and liable to fine and imprisonment at the discretion of the court trying the offense.

RECOMPENSE.

ARTICLE 5, SECTION 1. The recompense of the State Board of Health shall be, for the president, annually, $3500; the secretary, annually, $1800; but the remaining members will receive no recompense, except they shall have all traveling, hotel and other necessary expenses paid by the State while in active service for the State.

SECTION 2. All other employes of the board shall receive for their services, when on duty only, a sum not to exceed $3 per day, except that quarantine physicians may receive a sum of not less than $5 nor more than $10 per day, according to locality.

SECTION 3. The following sums shall be charged for inspecting and fumigating vessels at quarantine, when necessary: For every vessel of 2000 tons register or over, $20; for every vessel under 2000 and over 1000 tons, $10; for every vessel of 1000 tons or under, $5. These fees will cover certificate of health, if such should be required by the master of said vessels.

SECTION 4. For disinfecting trains: freight, $20; passenger, $10; to be paid by the owners or owner thereof.

SECTION 5. Any person who shall neglect or fail to obey any order of said Board of Health or its employes, when the same was served for the preservation of the public health, shall be guilty of a misdemeanor and upon conviction shall be fined not less than $5 nor more than $50 for each and every offense.

SECTION 6. The foregoing sums, charges and fines may be recovered, if necessary, by suit instituted by the Attorney-General of the State, and when so collected shall be turned over to the credit of the Health Department of the State.

SECTION 7. The sum of $40,000 yearly, for two years, or as much thereof as necessary, is hereby appropriated for the purpose of carrying out this act.

ARTICLE 6, SECTION 1. Resolved, that all laws and parts of laws heretofore existing in conflict with this act are hereby repealed, and the foregoing act in its entirety substituted.

